# Applicability of multiple quantitative ultrasound liver biomarkers in children and adolescents with severe obesity

**DOI:** 10.1186/s12887-025-05750-1

**Published:** 2025-05-16

**Authors:** Ivan Cetinic, Charlotte de Lange, Kerstin Lagerstrand, Jenny M. Kindblom, Lovisa Sjögren, Hanna Hebelka

**Affiliations:** 1 Department of Radiology, Sahlgrenska University Hospital, Region Västra Götaland, Gothenburg, Sweden; 2 Institute of Clinical Sciences, Sahlgrenska Academy, University of Gothenburg, Gothenburg, Sweden; 3 Department of Paediatric Radiology, Queen Silvia Children’s Hospital, Sahlgrenska University Hospital, Region Västra Götaland, Gothenburg, Sweden; 4 Department of Medical Physics and Biomedical Engineering, Sahlgrenska University Hospital, Region Västra Götaland, Gothenburg, Sweden; 5 Department of Paediatrics, Sahlgrenska University Hospital, Region Västra Götaland, Gothenburg, Sweden; 6 Department of Drug Treatment, Sahlgrenska University Hospital, Region Västra Götaland, Gothenburg, Sweden; 7 Institute of Medicine, Sahlgrenska Academy, University of Gothenburg, Gothenburg, Sweden

**Keywords:** Attenuation imaging, Elastography, Liver disease, Obesity, Steatosis, Ultrasonography, Inflammation, Fibrosis, Multiparametric, Ultrasound biomarkers

## Abstract

**Background:**

Obesity is associated with chronic liver disease, which is why improved non-invasive diagnostic assessment of liver affection is desirable. The ultrasound-based biomarkers Attenuation Imaging coefficient (ATI), Shear Wave Elastography (SWE), and Shear Wave Dispersion (SWD) have the potential to assess liver steatosis, fibrosis and inflammation/oedema respectively. The aim was therefore to evaluate the feasibility of applying ultrasound-based liver biomarkers in children and adolescents with severe obesity.

**Methods:**

Ultrasound was performed, before treatment, in 56 patients with childhood obesity (< 18 years) referred for bariatric surgery or treatment with glucagon-like peptide-1 receptor agonists. An ultrasound visualisation score (A: no limitations – D: severe limitations) was used. ATI, SWE and SWD were measured, irrespective of visualisation score, and compared to clinical data, serological measures and depth of measurement. Scan-rescan reproducibility measurements were performed, both for continuous measures using intraclass correlation coefficient (ICC) and for kappa coefficient using proposed reference thresholds for elevated/pathological values in children during fasting and free-breathing: > ATI 0.56 dB/cm/MHz, > SWE 4.9 kPa and > SWD 11.9 (m/s)/kHz.

**Results:**

The median (min–max) age of the 56 patients (51.8% male) was 16.2 years (9.9; 18) and the median BMI standard deviation score (SDS) was 4.4 (2.7; 7.3). The distribution of the visibility score was A 5.5%, B 50%, C 41% and D 3.5%. The median (min–max) ATI, SWE and SWD values were 0.58 dB/cm/MHz (0.32; 0.97), 7.2 kPa (4.3; 19.6) and 14.3 (m/s)/kHz (8.9; 24.3) respectively. Both ATI (β = -4.2; *r*^2^ = 0.3; *p* < 0.0001) and SWD (β = 0.14; *r*^2^ = 0.17; *p* = 0.0033) were influenced by depth of measurement. A weak association was found between ATI and serum triglycerides (β = 0.07; *r*^2^ = 0.12; *p* = 0.015). SWE was associated with BMI-SDS (β = 0.71; *r*^2^ = 0.09; *p* = 0.035). No other significant associations were found. ICC was moderate for ATI (0.61), fair for SWE (0.46) and fair for SWD (0.51). Kappa coefficient was substantial for ATI (0.77), excellent for SWE (1.0) and moderate for SWD (0.53).

**Conclusion:**

When accounting for visualization score, multiple ultrasound liver biomarkers appear applicable in most children and adolescents with severe obesity. Median ATI, SWE and SWD values were all increased, compared to currently known paediatric normal values. However, median ATI was likely underestimated due to depth dependence of measurement. Although caution is advised in clinical decision-making due to fair-moderate reproducibility between scans, most importantly, the biomarkers appear capable of differentiating between non-affected and affected liver in children with severe obesity.

## Background

According to the World Health Organization (WHO), up to 160 million children aged between five and 19 years live with obesity [1]. Obesity is defined using age- and gender-related body mass index (BMI) cut-offs. ISO-BMI, a modified BMI classification system developed by the International Obesity Task Force, accounts for growth and development in children and adolescents, making it suitable for paediatric populations. The definitions of overweight (ISO-BMI ≥ 25), obesity (ISO-BMI ≥ 30), severe obesity (ISO-BMI ≥ 35) and morbid obesity (ISO-BMI ≥ 40) are widely used [2, 3]. Obesity has become the most important driver of the increase in chronic liver disease in the paediatric and adolescent population, primarily due to it being highly associated with metabolic dysfunction-associated steatotic liver disease (MASLD) [4, 5]. A recent study investigating MASLD in Swedish patients with childhood obesity showed that MASLD was associated with a higher risk for developing youth-onset type 2 diabetes, further underlining the need for correct diagnosis [6].

Excessive fat in the liver leads to oxidative stress, which drives inflammation and progressive fibrosis [7]. Liver steatosis in children and adolescents with obesity has a high prevalence, up to 42% [4], and of these individuals, up to 62% are at risk of developing metabolic dysfunction-associated steatohepatitis (MASH) [8]. With the imminent risk of complications such as MASH, fibrosis and end-stage liver disease, detailed characterisation of the liver parenchyma is highly warranted in obese individuals for clinical decision-making, treatment and follow-up. The reference method for detailed liver characterisation is liver biopsy [9], but this is invasive, requires anaesthesia in most paediatric patients and is not suitable for monitoring disease. MRI is increasingly utilised for the diagnosis of fatty liver disease [10, 11]. However, the method is limited by its high cost, availability, and the fact that individuals with severe obesity may not always be suitable for MRI examination due to their body habitus. Alternative non-invasive methods are, thus, highly needed.

Modern ultrasound-based quantitative measurements of tissue echogenicity, stiffness and viscosity, obtained with Attenuation Imaging coefficient (ATI), 2D Shear Wave Elastography (SWE) and Shear Wave Dispersion (SWD), have been reported as promising tools for non-invasive tissue characterisation of the liver. Although not yet validated in a paediatric population, ATI has shown promise for reflecting liver fat content, with higher values indicating increased hepatic fat content [12, 13]. SWE provides an estimate of liver fibrosis by measuring liver stiffness [14], and SWD is a novel measure of viscosity which, according to a few studies, could mirror liver inflammation [15–17], although its clinical application is not yet known. The use of these markers could be of great value in discriminating the various components of liver affection in individuals with obesity non-invasively. However, a higher BMI is associated with greater body composition and often a concomitant reduction in ultrasound image visibility and quality, which limits the diagnostic yield of ultrasound in patients with obesity. Still, ultrasound is also used as a first-line imaging modality in this cohort, because it is cheap, accessible and non-ionising. Studies evaluating paediatric patients with severe obesity are however scarce [18, 19], and to our knowledge no studies exist with the application of ATI, SWE and SWD, either in children or adults living with obesity.

Considering the high risk of MASLD development in patients with paediatric obesity, and the potential benefit of being able to diagnose and estimate components of liver affection (steatosis/inflammation/fibrosis) non-invasively, the aim of the current study was to evaluate the feasibility of applying ultrasound with measurements of ATI, SWE and SWD in children and adolescents with severe obesity. A secondary aim was to report the range within which these markers lie in one such cohort, and to determine whether there is any association between the markers and serology measures.

## Material and methods

### Patient selection

From January 2023 and through May 2024, we consecutively recruited 56 children and adolescents (< 18 years), all of whom had been referred to the Regional Centre for Obesity at Queen Silvia Children’s Hospital in Gothenburg, Sweden, to this prospective cross-sectional study. All patients had been accepted for intervention in terms of either metabolic bariatric surgery or treatment with glucagon-like peptide-1 (GLP-1) receptor agonist, in addition to health behaviour and lifestyle treatment. However, at the time of the examination, all patients were solely treated with health behaviour and lifestyle treatment. The inclusion criteria were either obesity (ISO BMI > 30) with an obesity-related morbidity (such as type 2 diabetes, hypertension, polycystic ovary syndrome or hyperlipidaemia) or morbid obesity (ISO BMI > 40) without a co-morbidity. The exclusion criteria were inability to understand the language and inability to agree to informed consent or treatment initiation before the ultrasound examination.

### Ultrasound imaging procedure

**Fig. 1:**
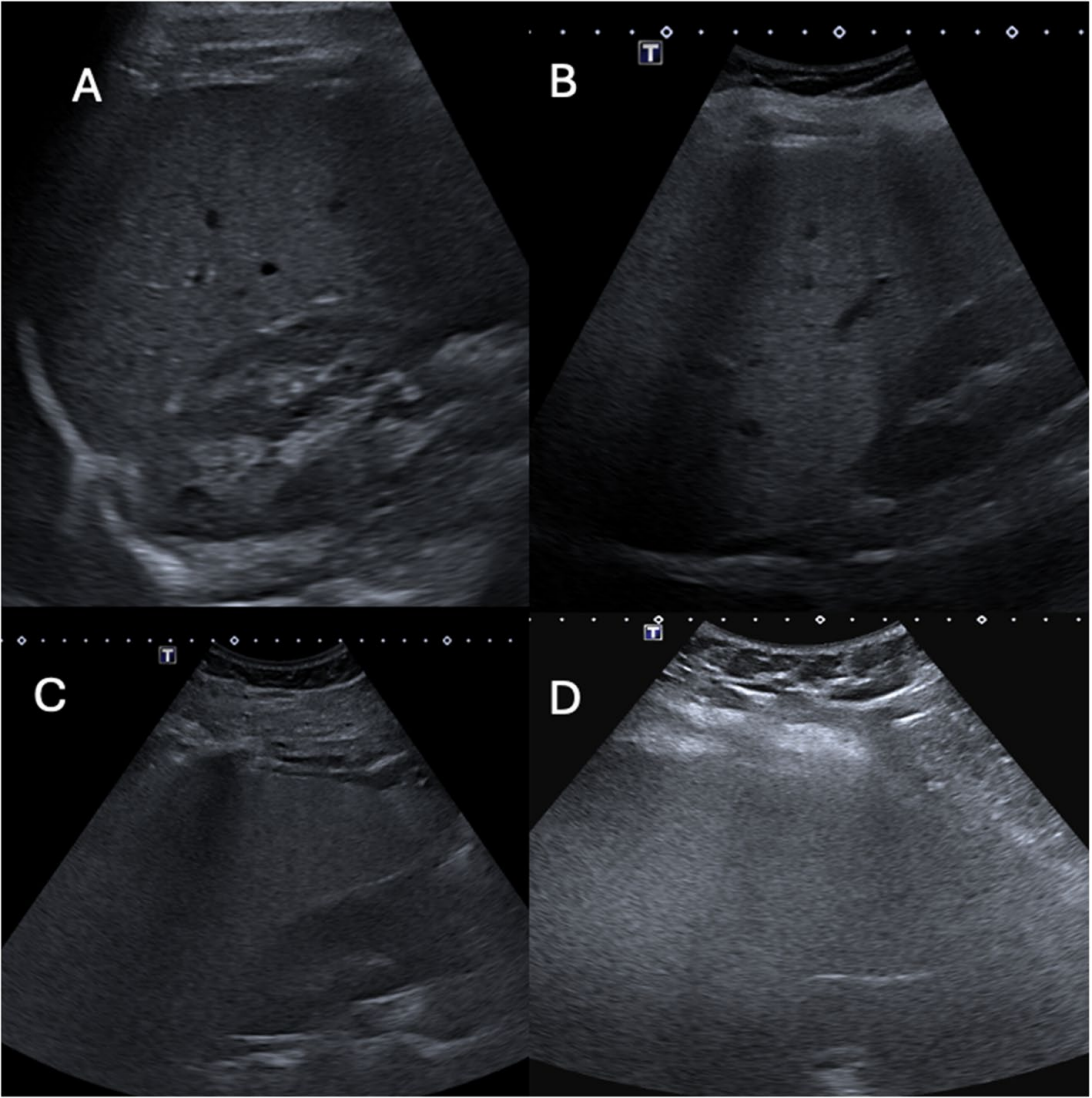
Visual representation of various visibility scores. **A** – liver is fully visualised; **B** – liver is visualised in near entirety; **C** – portions of liver diaphragm not visible; **D** – poor and non-diagnostic visualisation

All exams were conducted using a Canon Aplio i800 ultrasound machine (Canon, Tokyo, Japan) using the iC8x probe. Patients fasted for at least 4 h prior to the ultrasound. The examination was performed according to recommended standards, with the patient lying in a supine, slightly left-sided position with the right arm above the head [20, 21]. All patients were imaged while awake and freely breathing. The free-breathing technique was chosen since it is standard procedure at our children's hospital, supported by studies showing that breathing state does not significantly affect SWE, SWD and ATI values [22, 23]. Initially, a conventional grey-scale ultrasound examination of the liver was performed. To assess the quality and adequacy of the ultrasound examination, the Ultrasound Liver Imaging Reporting and Data System (US LI-RADS) [24] visualisation score was used with slight adaptation (Table 1, Fig. 1). Irrespective of visualisation score, all measurements were recorded.

**Table 1:** Modified Ultrasound Liver Imaging Reporting and Data System (US LI-RADS) visualisation score describing limitations in visualisation

Score	Criteria
A. No limitations	Liver homogenous and visualised entirely. No beam attenuation
	Doppler measurements of vena porta easily attainable
B. Minimal limitations	Minimal beam attenuation or shadowing
	Liver visualised in near entirety
	Doppler measurements of vena porta easily attainable
C. Moderate limitations	Moderate beam attenuation or some portions of liver or diaphragm not visualised
	Doppler measurements of vena porta attainable
D. Severe limitations	Majority (> 50%) of liver not visualised. Majority (> 50%) of diaphragm not visualised
	Doppler measurements of vena porta not possible

For ATI measurements, images of the right liver lobe were acquired via intercostal approach, 1.5–3 cm below the liver capsule, using continuous mode, taking care not to use transducer compression. Five distinct ATI measurements (dB/cm/MHz) were recorded, and the median value was used for analysis [20].

For SWE measurements, a 1-cm-diameter region of interest (ROI) was placed in the right lobe of the liver through an intercostal approach. Ten SWE measurements (kPa) were obtained, with the median of these registered for analysis.

Since SWD measurements are derived from the shear waves generated and measured by SWE, the SWD measurements are obtained simultaneously with SWE measurements, using the same technique. The median of ten SWE measurements [(m/s)/kHz] was used for analysis.

Exclusion criteria for ATI measures were suboptimal measurements, i.e., a measurement of *R*^2^ < 0.8. For SWE and SWD measurements, exclusion criteria were examinations with technical difficulties (such as kPa interquartile range (IQR)/median > 30%) [21].

### Depth of measurement

For all quantitative measures, the ultrasound machine automatically recorded the distance from the skin to the region of interest (ROI) box. Towards the end of data sampling for the study, indications that depth of measurement could impact ATI values were published [25]. Therefore, to assess the impact of depth on ATI, we also measured the distance from the skin to the liver capsule and from the liver capsule to the upper border of the ROI box retrospectively.

### Ultrasound operators and scan-rescan reproducibility

All initial ultrasounds were performed by one of two radiologists (HH and CL) with at least five years'experience in elastography ultrasound and > 20 years'experience in diagnostic ultrasound. A third radiologist (IC), with approximately one year's experience in elastography technique, was involved in evaluating scan-rescan reproducibility. Operators were blinded to biochemical markers and any prior imaging results.

All patients (*n* = 56) underwent the initial grey-scale ultrasound and quantitative evaluation of ATI, SWE, and SWD. A subset of 19 patients (34%) had a second ultrasound immediately following the first by one of the three radiologists to evaluate scan-rescan reproducibility. These examinations included visualisation score and measures of ATI, SWE and SWD, and were performed blinded to the outcome of the first ultrasound.

Scan-rescan reproducibility was evaluated both for continuous measures, using intraclass correlation coefficient (ICC), and for categorical measures, using kappa coefficient with the following cut-off values for elevated/pathological values: > ATI 0.56 dB/cm/MHz, > SWE 4.9 kPa and > SWD 11.9 (m/s)/kHz [14, 26]. Awaiting proper validation, values below these thresholds have been reported, using the same ultrasound machine (Toshiba/Canon/Aplio i800), likely excluding significant pathology in children during fasting and free-breathing [14, 26].

### Clinical data and serological testing

Clinical data including sex, age, weight, height, BMI and BMI-SDS was recorded within 30 days of the ultrasound examination. Blood samples were taken within a window of 12 months prior to 30 days after the ultrasound and before any treatment initiation. The following serology tests were sampled: glucose, HbA1c, insulin, cholesterol, triglycerides, high-density lipoprotein (HDL), low-density lipoprotein (LDL), C-reactive protein (CRP), aspartate transaminase (AST), alanine transaminase (ALT), platelets and white cell count.

### Statistical analysis

All statistical analysis was performed using SAS software version 9.4 (Cary, NC, USA). The results are presented as median (min–max) for continuous variables, since many variables were not normally distributed, and n (%) for categorical variables. The variables were diagnosed by inspecting histogram and Q-Q plots, identifying whether or not the variables approximate normality. Univariable linear regression was used to estimate how ATI, SWE and SWD relate to serological blood work and depth. The results are given as beta (slope of the linear regression) with 95% confident interval (CI), *r*^2^ how much of the variance in ATI, SWE and SWD will be explained by the linear predictor, and *p*-value. ICC was calculated using Shrout-Fleiss analysis, with random models, for continuous repeatability measures, and Bland–Altman plots were used to display agreement. Cicchetti’s scheme was used to interpret the coefficients, with < 0.40 indicating poor repeatability, 0.40–0.59 indicating fair repeatability, 0.60–0.74 indicating good repeatability and 0.75–1 indicating excellent repeatability [27]. Simple kappa coefficients were used for scan-rescan repeatability measures using cut-off values for SWE, SWD and ATI, with Landis’ scheme [28] used for interpretation, where a kappa between 0.6 and 0.8 was considered substantial agreement and > 0.80 almost perfect agreement. All significance tests were two-sided, and were conducted with 5% as the significance level.

## Results

Demographics and clinical data, quantitative liver biomarkers and serological bloodwork are detailed in Tables 2 and 3. ATI was measured in all included patients (*n* = 56), but we excluded measurements of SWE and SWD in seven (13%) patients due to IQR/median > 0.30 kPa. In the scan-rescan reproducibility measurements, we excluded measurements of SWE and SWD in one patient for the same reason. Three patients (5.5%) had no visual limitations during the ultrasound exam as in visualisation score of A, 28 (50%) patients had score B, 23 (41%) had score C and 2 (3.5%) had score D.

**Table 2:** Demographic clinical data and serological blood work

Clinical data	Study participants
Male	29 (51.8%)
Female	27 (48.2%)
Age (year)	16.2 (9.9; 18) 15.6 (2.0), *n* = 56
Height (cm)	171.6 (10.5) 172 (144.8; 195.7), *n* = 55
Weight (kg)	121.0 (24.8) 122.5 (55; 198.7), *n* = 55
BMI	41.0 (5.8) 40.7 (26.1; 59.8), *n* = 55
BMI-SDS	4.59 (0.94) 4.44 (2.72; 7.32), *n* = 54
Surgical treatment	22 (39%)
Medical treatment	34 (61%)
Serology	
Glucose (mmol/L)	5.52 (0.52) 5.4 (4.6; 7.2) (5.36; 5.68), *n* = 45
HbA1c (mmol/mol)	33.9 (5.1) 33.5 (9.6; 45) (32.4; 35.4), *n* = 46
Insulin (mIU/L)	31.1 (23.2) 25 (9; 120) (24.3; 37.9), *n* = 47
Cholesterol (mmol/L)	4.27 (0.93) 4.1 (2.4; 6.8) (4.00; 4.54), *n* = 48
Triglycerides (mmol/L)	1.38 (0.63) 1.3 (0.59; 3.3) (1.20; 1.57), *n* = 47
HDL (mmol/L)	1.13 (0.36) 1.1 (0.68; 2.6) (1.02; 1.24), *n* = 47
LDL (mmol/L)	3.02 (1.00) 2.85 (1.1; 5.7) (2.73; 3.31), *n* = 48
CRP (mg/dL)	7.78 (7.60) 5.7 (0.5; 31) (5.50; 10.07), *n* = 45
WBC (× 10*9/L)	7.78 (2.42) 7.85 (3.9; 15.8) (7.07; 8.50), *n* = 46
PLT (× 10*9/L)	304.3 (73.8) 287 (192; 543) (282.4; 326.2), *n* = 46
AST (µkat/L)	0.62 (0.39) 0.52 (0.19; 2.42) (0.50; 0.73), *n* = 47
ALT (µkat/L)	0.90 (1.01) 0.63 (0.17; 6.75) (0.61; 1.12), *n* = 48

For continuous variables, mean (SD)/median (min; max)/(95% confidence interval for mean)/*n* = is presented. *BMI* body mass index, *SDS* standard deviation score, *HDL* high-density lipoprotein, *LDL* low-density lipoprotein, *CRP* C-reactive protein, *WBC* white blood cell, *PLT* platelets, *AST* aspartate aminotransferase, *ALT* alanine aminotransferase

**Table 3:** Liver biomarkers and depth measurements

	**Study population**
ATI (dB/cm/MHz)	0.60 (0.14) 0.58 (0.32; 0.97) (0.56; 0.64), *n* = 56
SWE (kPa)	7.97 (3.08) 7.2 (4.3; 19.6) (7.09; 8.86), *n* = 49
SWD (m/s)/kHz	14.4 (3.0) 14.3 (8.9; 24.3) (13.5; 15.3), *n* = 49
Depth SWE and SWD (cm)	6.0 (1.06) 5.9 (4; 9.2), *n* = 49
ATI liver capsule to ROI (cm)	1.90 (1.1) 2.22 (1; 3,5), *n* = 56
ATI skin to liver capsule (cm)	3.74 (0.96) 3.75 (1.5; 6), *n* = 56
ATI depth total (cm)	5.70 (0.98) 5.5 (3.5; 8.5), *n* = 56

For continuous variables, mean (SD)/median (min; max)/(95% confidence interval for mean)/*n* = is presented. *ATI* attenuation imaging, *SWE* shear wave elastography, *SWD* shear wave dispersion, *ROI* region of interest

### Attenuation imaging

There was a significant but very weak negative association between ATI and BMI-SDS (β = −0.05; *r*^2^ = 0.14; *p* = 0.006). A weak positive association was seen between ATI and serum triglycerides (β = 0.07; *r*^2^ = 0.12; *p* = 0.015). No other significant associations between ATI and analysed parameters could be found (Table 4).

**Table 4:** Associations between ultrasound biomarkers and clinical data and serology tests

Variable	ATI (dB/cm/MHz)			SWE (kPa)			SWD (m/s)/kHz		
	Beta (95% CI)	*p*	*R*^2^	Beta (95% CI)	*p*	*R*^2^	Beta (95% CI)	*p*	*R*^2^
BMI	−0.01 (−0.02;−0.00)	0.0007	0.20	0.12 (−0.01;0.25)	0.066	0.07	0.09 (−0.06;0.25)	0.22	0.03
BMI SDS	−0.05 (−0.09;−0.02)	0.0060	0.14	0.71 (0.05;1.37)	0.035	0.09	0.56 (−0.35;1.48)	0.22	0.03
AST (ukat/L)	0.06 (−0.05;0.17)	0.28	0.03	−1.92 (−4.08;0.23)	0.079	0.08	−1.78 (−3.63;0.08)	0.060	0.09
ALT (ukat/L)	0.01 (−0.03;0.05)	0.56	0.01	−0.56 (−1.39;0.28)	0.19	0.04	−0.34 (−1.09;0.40)	0.36	0.02
HbA1c (mmol/mol)	0.00 (−0.01;0.01)	0.60	0.01	−0.20 (−0.36;−0.04)	0.015	0.13	−0.03 (−0.18;0.12)	0.67	0.00
Cholesterol (mmol/L)	0.03 (−0.01;0.07)	0.17	0.04	−0.24 (−1.20;0.73)	0.62	0.01	0.12 (−0.73;0.98)	0.77	0.00
Triglyceride (mmol/L)	0.07 (0.02;0.13)	0.015	0.12	−0.88 (−2.26;0.50)	0.20	0.04	0.23 (−0.99;1.46)	0.70	0.00

The β value for each independent variable indicates the expected change in the dependent variable for a one-unit increase in that independent variable, assuming all other variables in the model are held constant. *P* probability value, *BMI* body mass index, *SDS* standard deviation score, *AST* aspartate aminotransferase, *ALT* alanine aminotransferase. *CI* confidence interval, *ATI* attenuation imaging, *SWE* shear wave elastography, *SWD* shear wave dispersion

### Shear wave elastography and shear wave dispersion

SWE showed a significant association with BMI-SDS (β = 0.71; *r*^2^ = 0.09; *p* = 0.035) and a weak negative association with HbA1c (β = −0.20; *r*^2^ = 0.13; *p* = 0.015). No other significant associations between SWE and analysed parameters could be found. SWD showed no association with any of the serum biomarkers (Table 4).

### Depth

**Fig. 2:**
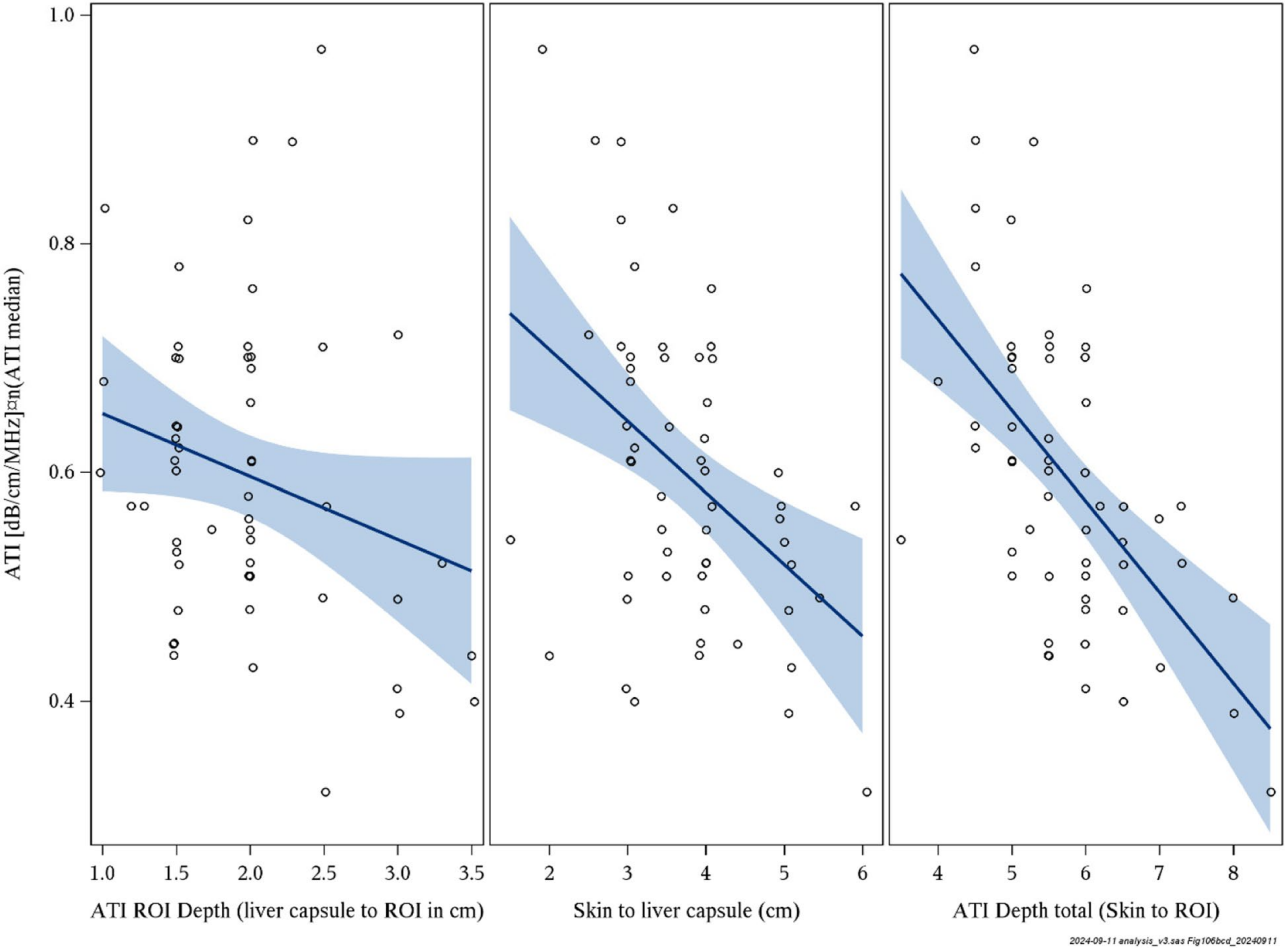
Depth dependence of attenuation imaging

There was a significant association between ATI and distance from skin to ROI box (β = −4.2; *r*^2^ = 0.33; *p* < 0.0001) and from skin to liver capsule (β = −3.1; *r*^2^ = 0.19; *p* = 0.0007), but not between ATI and distance from liver capsule to ROI (β = −1.07; *r*^2^ = 0.06; *p* = 0.071). SWD was slightly influenced by the distance from the skin to ROI (β = 0.14; *r*^2^ = 0.17; *p* = 0.003), while SWE did not show any association (β = 0.09; *r*^2^ = 0.07; *p* = 0.064) (Fig. 2).

When stratifying by obesity categories; obesity (ISO-BMI ≥ 30), severe obesity (ISO-BMI ≥ 35), and morbid obesity (ISO-BMI ≥ 40), and reanalysing the association between ATI and the distance from the skin to the ROI box, a significant association was observed only for ISO-BMI ≥ 40 (*n* = 32; β = −6.19; *r*^2^ = 0.40; *p* < 0.0001). No significant associations were found for ISO-BMI ≥ 30 (*n* = 4; β = −0.90; *r*^2^ = 0.03; *p* = 0.84) or ISO-BMI ≥ 35 (*n* = 17; β = −2.90; *r*^2^ = 0.21; *p* = 0.064).

### Scan-rescan reproducibility measurements

**Fig. 3:**
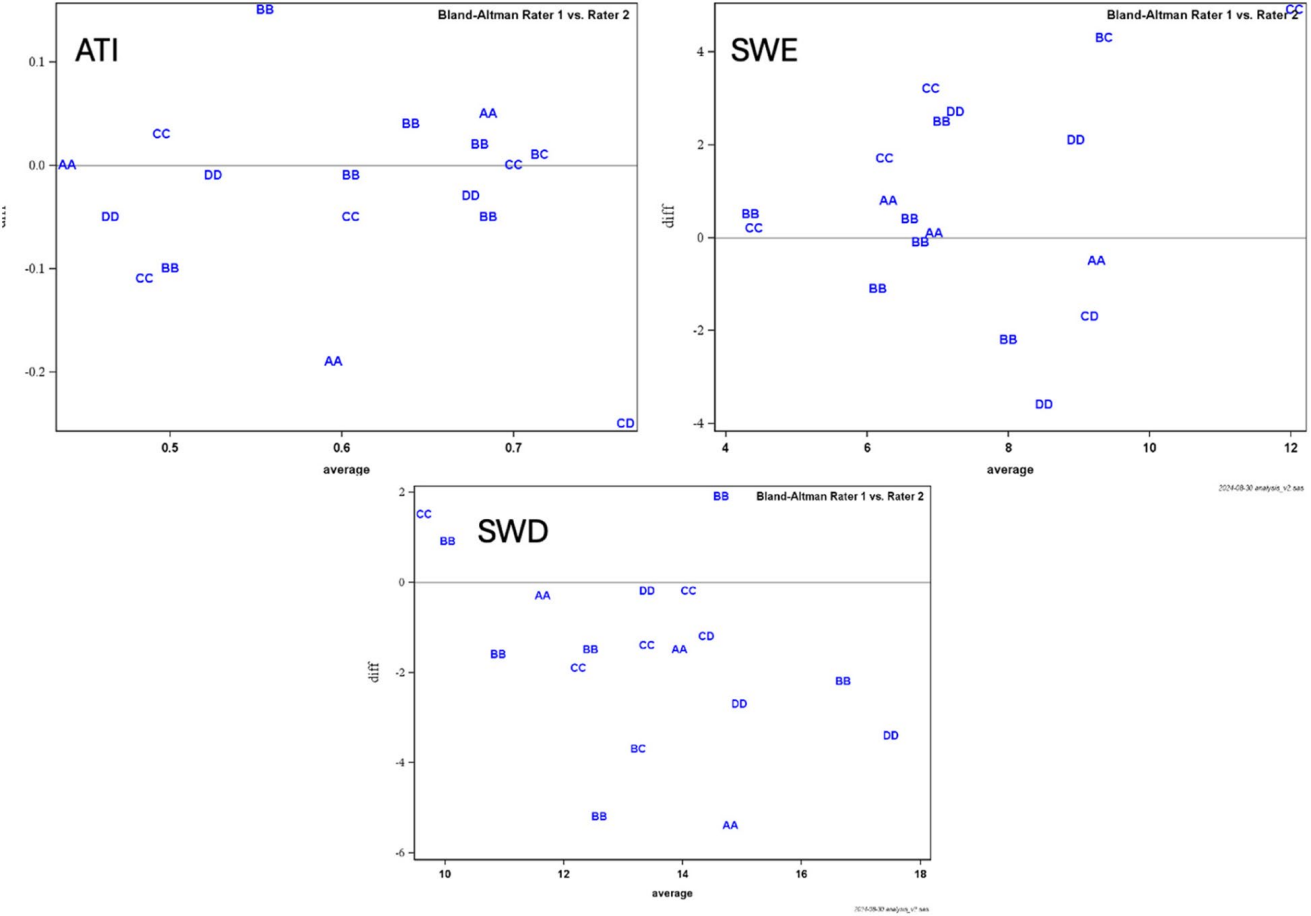
Bland–Altman plots comparing differences in measurements of ATI, SWE and SWD between raters. The letters represent visualisation scored by the two raters (**A** – excellent, **B** – good, **C** – moderate, **D** – poor)

ATI measurements showed moderate reproducibility with ICC 0.64 (coefficient of variance 11%). SWE showed fair reproducibility with ICC 0.46 (coefficient of variance 22%), and SWD showed fair reproducibility with ICC 0.51 (coefficient of variance 13%). Bland–Altman plots of differences in measurements across ATI, SWE and SWD are detailed in Fig. 3. Kappa coefficients for distinguishing between normal/pathological using cut-off values were substantial for ATI (k = 0.77), excellent for SWE (k = 1.0) and moderate for SWD (k = 0.53).

## Discussion

This study investigated the feasibility of applying ultrasound with the markers ATI, SWE and SWD in children and adolescents with severe obesity, and found it applicable in the majority of individuals. Image visibility and quality were found to have no limitations, or only minor limitations, in over 50% of patients. Median ATI, SWE and SWD were above currently known paediatric normal values, indicating various forms of liver affection [14, 29].

Despite the expected difficulty of assessing the liver with ultrasound in patients with severe obesity, 56% of the patients had no or minimal limitation (visualisation scores of A or B respectively) and 41% had moderate limitation (a score of C), and according to these results it is therefore worth trying to assess the liver with ultrasound despite very high BMI (up to 59.8 in this study). With moderate limitation in visualising the liver, detection of focal liver lesions can be hampered, which is a drawback. However, visual subjective estimation of increased liver echogenicity, as well as measurement of ATI, SWE and SWD, was possible in most cases. The inclusion of challenging patients with moderate to poor visualisation (scores of C and D) may partly explain the fair to moderate ICC values. For example, looking at the Bland–Altman plot for SWE (Fig. 3B), the concordance between raters was better in most cases when visualisation was not hampered with moderate to major limitations. To address this, we reanalysed the ICC after excluding examinations with a visualization score of C or D; however, this did not result in improved ICC values. Therefore, suboptimal visualization alone did not fully explain the limited reproducibility. Additional factors, such as the limited experience of one observer and the complexity of the cohort, characterized by increased abdominal circumference, likely also influenced the measurements, even when visualization was adequate. The fair to moderate scan-rescan reproducibility coefficients highlights important limitations when interpreting ATI, SWE, and SWD measurements in clinical practice for paediatric patients with severe obesity. While these biomarkers offer valuable alternatives to liver biopsy, our findings suggest that single measurements should be interpreted with caution and not used in isolation for clinical decision-making. We recommend being careful when using the biomarkers for clinical decision making in patients with a score of C and avoiding measurements in patients with a score of D. The biomarkers should be used as complementary tools alongside conventional imaging findings, biochemical markers, and clinical assessment, with serial measurements being preferable to establish individual baselines rather than relying solely on absolute threshold values.

**Fig. 4:**
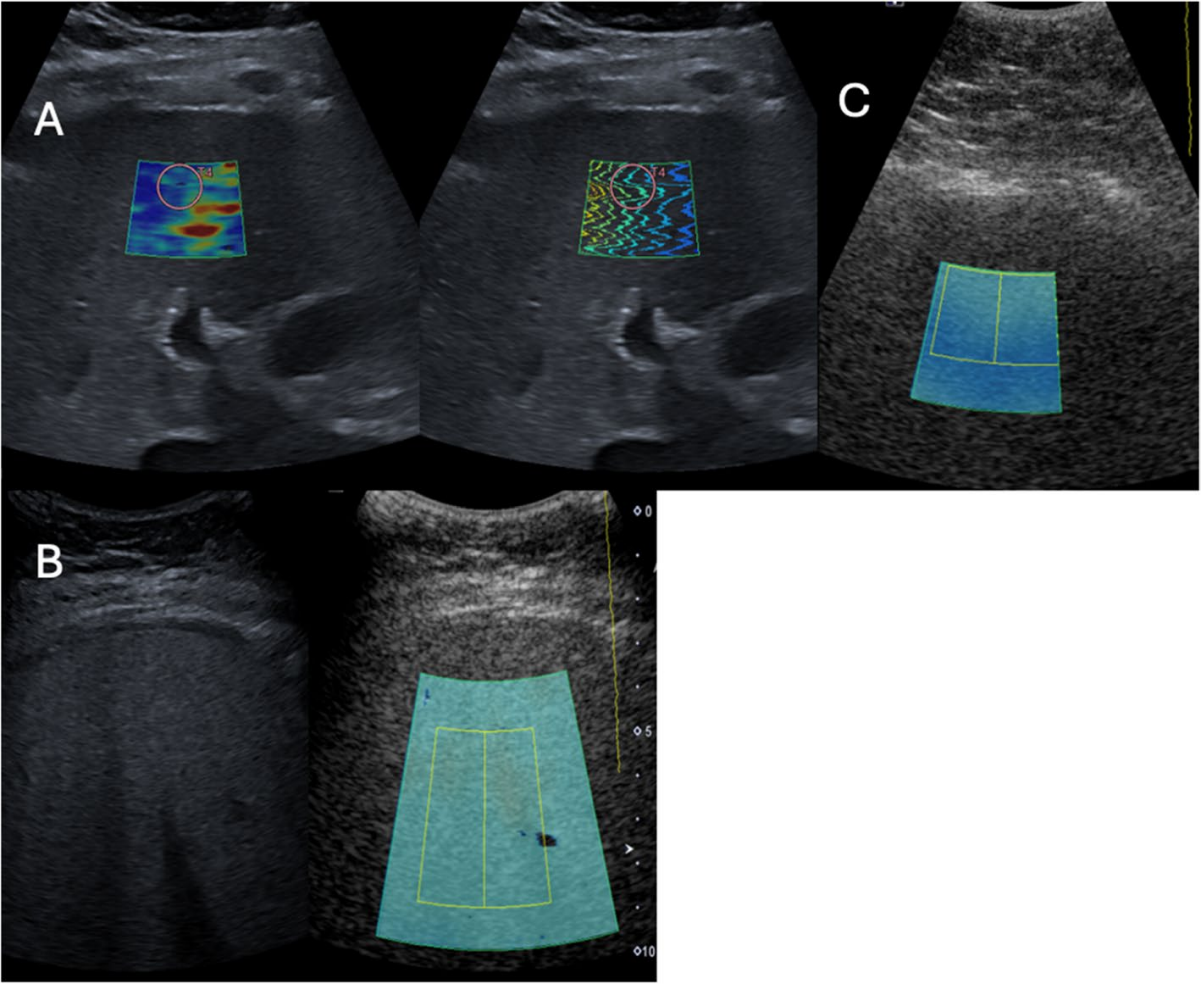
**A** Patient with good visibility (score B) and increased levels of SWE at 11.6 kPa, suggestive of fibrosis; **B** Patient with visibility score C and an increased ATI measurement (0.84 dB/MHz/cm), suggestive of high grade steatosis; **C** Patient with visibility score D and low ATI score (value 0.32 dB/MHz/cm). As can be seen in patient C, ATI measurement is not adequate, with poor B-mode image and inadequate placement of the ROI box, including dark blue areas. The poor visualisation makes it impossible to obtain adequate placement of ROI box, hence inadequate measurement of ATI

Despite a cohort with severe obesity, the median ATI value was only 0.58 dB/cm/MHz, which is slightly above, or in parity with, the mean values reported in prior studies on healthy children [16, 26, 29]. However, there was a large variation, with increased values in several patients (Fig. 4B) suggesting various grades of steatosis within the cohort. Additionally, a suboptimal visualisation score (Fig. 4C) and high distance from skin to ROI are likely confounders that could have led to an underestimation of steatosis. A recent study by Ferraioli et al. [25] showed that ATI values decreased by 0.052 dB/cm/MHz units per cm increase in depth between the liver capsule and the upper edge of the ROI box, and that these values were also affected by the skin-to-liver capsule distance. This was not known when performing the current study, and was hence not accounted for during data sampling. The dependence on depth for ATI values (Fig. 2) was however confirmed, to our knowledge, for the first time in a paediatric cohort. The depth dependence would largely explain the unexpected weak but negative association found between ATI and both BMI and BMI-SDS, which otherwise does not seem logical. This is further supported by our results, where a subanalysis stratified by ISO-BMI categories (obesity, severe obesity, and morbid obesity) demonstrated that a significant negative association between ATI and distance skin to ROI box was only observed in the morbid obesity group. Even if not standardised for depth, the median distance of 2 cm between liver capsule and ROI in the current cohort is within the suggested optimal range [25], as is the median total depth of 5.5 cm. This, lack of significant depth dependence in groups without morbid obesity and the shown significant association between triglycerides and ATI, implies that the obtained ATI measures in the current study are not random. However, in some cases with poor visualization and/or a large distance to the ROI, the measurements are likely irrelevant and underestimated (Fig. 4C).

A depth dependence was also shown for SWD, with values slightly increasing with depth. This has not been reported previously, and contradicts two phantom studies showing that SWD measurements decrease with increasing depth [30, 31]. Only SWE did not show a significant depth reliance. These results demonstrate that a standardised depth-based approach is necessary to minimise confounding factors when using these ultrasound markers on the liver.

Even though liver biopsy is regarded as a reference method for liver characterisation, magnetic resonance imaging with proton density fat fraction (MRI-PDFF) has been used in recent years as a sufficient reference method for liver fat quantification [32, 33], with the advantage of being non-invasive and enabling fat estimation of the entire liver. However, the drawbacks are poor MRI accessibility and possible anaesthesia requirement in this cohort. Additionally, gantry size and maximum table load can be limiting factors in patients with severe obesity. Several studies in adults have shown ATI to correlate well with MRI-PDFF in fat quantification [34, 35], and similar results have been shown for liver fat assessment from other vendors [32, 33]. Ultrasound would therefore be a convenient alternative for monitoring any increase or decrease in steatosis. In progressive liver disease, it is known that while fibrosis increases, liver fat content decreases [36], and a consequent reduction in liver fat does not necessarily imply an improved condition. Thus, imaging these high-risk patients should ideally include fibrosis evaluation. SWE is a validated method for fibrosis estimation in adults, and several studies are also showing promise in the paediatric group [14, 17, 26]. Even though no SWE cut-off values have been established in children, a few recent studies on normative values suggest that values below 4.9 kPa rule out fibrosis [16, 17, 26, 29]. The median value of 7.2 kPa (Table 3) in our cohort suggests the presence of fibrosis, and can be a decisive parameter for treatment, prognosis and mortality overall [8, 36]. SWD also showed a slightly increased median value in our cohort at 14 (m/s)/kHz (Table 3) compared to previous studies, suggesting a cut-off value at 11–12 (m/s)/kHz for healthy children and adolescents [15, 16, 26, 29]. The increased median SWD value, with a wide range (8.9 (m/s)/kHz to 24.3 (m/s)/kHz), suggests various grades of inflammation within the cohort. However, since it was found that SWD slightly increases with increasing depth, further research is needed to grasp the full clinical implication and interpretation.

The inherent challenges of examining a cohort with severe obesity, including a relatively inexperienced rescan radiologist, have likely impacted our fair to moderate repproducibility estimates. Further, we included results from all measurements, even those with score D that we do not recommend using these ultrasound markers on (Fig. 1D). Despite the fair reproducibility regarding SWE, the coefficient of variation was 22%, which means that if, for example, 8 kPa is obtained, the margin of error lies between 6.2 and 9.8, which still is pathological. Could this be enough to distinguish diseased from healthy liver tissue in the current cohort (Fig. 4A)? The perfect agreement to differentiate between SWE values below or above 4.9 kPa strengthens this argument. ATI showed a substantial kappa agreement using cut-off values, but its reliance on depth needs to be taken into account. It would therefore be recommended to use the same depth distance in longitudinal monitoring to decrease confounders.

### Limitations

Due to the lack of a valid reference method, i.e., liver biopsy, it is unknown whether abnormal ATI, SWE and SWD values resulted from inherent liver affection. However, although ATI, SWE and SWD still need validation in paediatric cohorts, several studies have compared them with biopsy-correlated results, demonstrating that these methods do reflect liver impairment [17, 37, 38]. In addition to the reproducibility limitations discussed above, it is not unlikely that the examiner could have unintentionally used probe pressure to get a better field of view to compensate for impaired visualisation in patients with large body composition. Some studies have shown that the use of free-breathing could cause a higher variability in values [39], while others have shown that free-breathing does not have a significant effect [23]. Since MASLD is emerging in younger age groups, we decided to use free-breathing for the purpose of clinically applicability in children of all ages. Finally, our serological blood work was up to 12 months old in certain cases, which can be deemed to be outdated, but the decision to include these samples was taken in collaboration with our clinicians.

## Conclusion

When accounting for visualisation score, multiple ultrasound liver biomarkers seem applicable in the majority of children and adolescents living with severe obesity. Median ATI, SWE and SWD values were all increased, compared to currently known paediatric normal values. However, median ATI was likely underestimated due to the depth dependence of measurement. The observed suboptimal reproducibility indicates that caution must be taken when using the biomarkers'absolute values for clinical decision-making. Most importantly, if accounting for visualisation score and standardising depth of measurement, it seems that these ultrasound biomarkers can at least be used to differentiate between non-affected and affected liver tissue in severely obese children and adolescents.

## Data Availability

No datasets were generated or analysed during the current study.

## Abbreviations

SWE: Shear wave elastography
SWD: Shear wave dispersion
ATI: Attenuation imaging
WHO: World Health Organisation
MASLD: Metabolic dysfunction-associated steatotic liver disease
MASH: Metabolic dysfunction-associated steatohepatitis
BMI: Body mass index
BMI-SDS: Body mass index standard deviation score
GLP-1: Glucagon-like peptide-1
US LIRADS: Liver Imaging Reporting Data System
ICC: Intraclass correlation coefficient
MRI PDFF: Magnetic resonance proton density fat fraction
IQR: Interquartile range
ROI: Region of interest
HDL: High-density lipoprotein
LDL: Low-density lipoprotein
CRP: C-reactive protein
WBC: White blood cell
PLT: Platelets
AST: Aspartate aminotransferase
ALT: Alanine aminotransferase
